# Many faces of bankers' identity: how (not) to study dishonesty

**DOI:** 10.3389/fpsyg.2015.00302

**Published:** 2015-03-18

**Authors:** Marek A. Vranka, Petr Houdek

**Affiliations:** ^1^Faculty of Arts, Charles UniversityPrague, Czech Republic; ^2^Faculty of Social and Economic Studies, J. E. Purkyne UniversityUsti nad Labem, Czech Republic; ^3^Center for Behavioral Experiments (CEBEX)Prague, Czech Republic; ^4^Faculty of Business Administration, University of EconomicsPrague, Czech Republic

**Keywords:** dishonesty, identity priming, labeling, banking culture, stereotype, entitlement, money priming, experimental methodology

## Introduction

In a recent Nature article, Cohn et al. (2014, henceforth CFM) boldly claim that their results “suggest that the prevailing business culture in the banking industry weakens and undermines the honesty norm” (p. 1).

The main empirical finding of the CFM paper is that a group of bank employees that answered a series of questions about their work subsequently reported higher proportion of wins in a coin flipping task (58.2%) than a control group of bank employees (51.6%) that answered questions of a similar form but focused on their leisure activities instead of banking. Because one can on average correctly guess the outcome of only half of the flips, a higher rate of success reported by a group indicates that some of its members cheated. From this result the authors infer that asking bankers about their work makes their professional identity more salient, which leads them to cheat more. That in turn is supposed to suggest that “prevailing business culture in the banking industry favors dishonest behavior” (CFM, p. 3).

We do not want to question the reality of their empirical findings. However, in line with previous critiques of priming research (Stafford, 2014), we want to point out certain limitations of the inferences which can be drawn from the empirical results: First, the observed dishonest behavior after the banking identity prime does not necessarily mean that there are social norms encouraging dishonesty in the banking industry. Instead of norms, a negative stereotype of bankers' dishonesty might be responsible for the observed effect: other stereotypes were shown to influence behavior—for example priming of the “hooligan” stereotype decreases performance in a trivia quiz (Dijksterhuis and van Knippenberg, 1998). Second, it is not even necessary that the observed difference in cheating had been caused by the priming of the professional identity. Either the other group could have been primed to cheat less, or other related concepts could have been primed in the experimental condition together with the banking identity and caused more cheating.

Our goal is to highlight the fact that the study of cultural norms on behavior using indirect methods often precludes us from making strong conclusions, because alternative explanations are plentiful and hard to rule out. We offer suggestions how future studies can build on the CFM design and more reliably answer the question whether the social norm in banking really supports dishonesty.

## Dishonest culture or just a label

The most general issue with the interpretation of the results in CFM is that even if the primed banking identity really had caused more cheating, one would still not be able to conclude that the norms in the banking industry are responsible. Non-banker participants in one of the authors' surveys believed “that bank employees would be the most dishonest group” (CFM, p. 3). Because it is plausible that bankers themselves are well aware of this bad reputation, it is also feasible that bankers did not behave accordingly to the norm intrinsic to the banking culture itself, but accordingly to the societal expectations for their behavior (Baay et al., 2014) when their identity as bankers was made salient. Interestingly, in a similar study with a prison population, two CFM authors recognize this possibility: “a person's identity can change if society treats him or her as a criminal, leading to the adoption of behavioral propensities consistent with the criminal label” (Cohn et al., unpublished, p. 1, footnote 2).

Previous studies have shown that performance in cognitive ability tasks could be lowered (Steele and Aronson, 1995; Spencer et al., 1999) or increased (Shih et al., 1999) by priming of gender or racial identity. These results from the “stereotype threat” literature are not interpreted as proofs of existence of social norms for higher or lower cognitive abilities of women or Asians. Instead, it is proposed that societal expectations are causing the observed changes in the task performance. One possible explanation of the effect is that when a stereotype that one is inferior in a given task is primed, one is not motivated to perform well (Dijksterhuis and van Knippenberg, 1998). Similarly, bankers might not be motivated to engage in “costly” honest behavior, when they would regardless be perceived as dishonest by others.

The bad reputation of bankers could have been caused by extreme, publicized cases of fraud that had created a false information cascade (Bikhchandani et al., 1998; Anderson et al., 2006; Hoff and Pandey, 2006). However, the stereotype would lead to the same observed increase of dishonest behavior even if no dishonesty-supporting social norms existed in the banking industry. Therefore, the main conclusion of CFM about the existence of such norms is not fully warranted.

## Alternative causes of the observed difference in dishonesty

**Figure 1:**
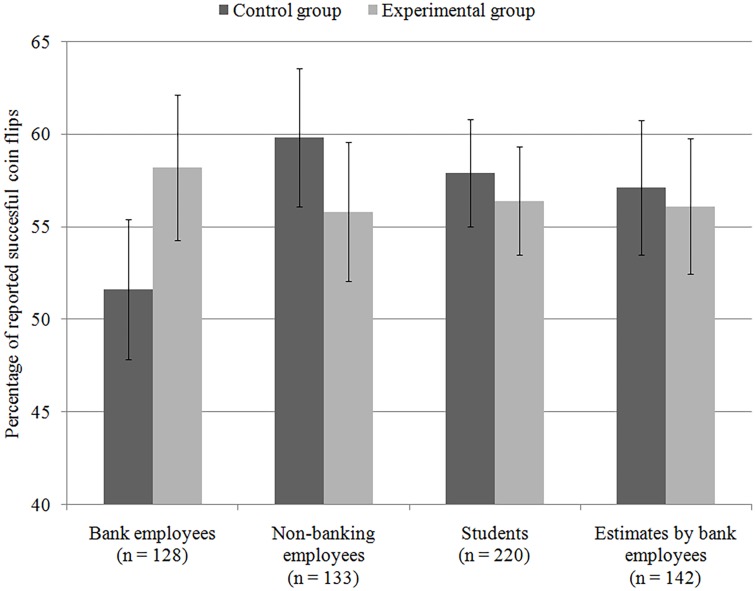
**Dishonesty rates in different samples**. Error bars indicate 95% CIs. Figure adapted from the CFM results.

The main empirical result of CFM is that bankers answering work-related questions cheated significantly more than those answering questions about their free time. However, from the look at the rates of cheating in additional CFM experiments using the same design but different participants (see Figure 1), it becomes clear that this finding could be interpreted in a completely opposite way: the questions about their leisure activities caused bankers to cheat less. This interpretation is at least as plausible as the one the authors chose. However, it could have been masked by calling the group with questions about leisure activities a “control group.” This implies that it represents a neutral baseline with which the effect of experimental manipulation can be compared. But there are no truly neutral questions, as everything could make some concept salient and thus influence the subsequent behavior. This is why more than one “control group” should be used and only when the expected difference between experimental and control conditions is observed for all of them, we can consider the causal effect of experimental manipulation proven.

Both above mentioned arguments hold even if we assume that the professional identity (and nothing else) was successfully primed in the experimental condition. Below, we argue that although the authors made a decent attempt to verify whether the bankers' identity had been primed, the used manipulation task could not completely rule out the possibility that some other concepts had been primed as well and subsequently caused the observed difference in cheating.

In the manipulation check task, bankers in the experimental condition completed word fragments with banking-related words more often than participants in the control group. However, this result means only that banking-related words were more activated for the participants who had just finished answering questions about banking. The activation of other related concepts was not measured and therefore the possibility of alternative causes of the difference in cheating remains open.

One possibility is that the difference was caused by priming of the concept of money (Vohs et al., 2008). The observed stronger agreement with a statement that status is determined by financial success is also in line with this possibility, as bankers with more strongly activated concept of money could agree with it more and also cheat more. However, the propensity to be primed with money and/or the influence of this prime on a subsequent behavior is likely to be a personal characteristic (Smeesters et al., 2009; Furnham et al., 2014) and not a feature of the banking culture.

The authors themselves discuss the possibility of the money priming and claim it was ruled out in a control experiment, in which students in an experimental group were asked to answer hypothetical questions about work in the banking sector and their rate of misreporting was not statistically different from the control condition. The authors claim that if the concept of money was responsible for cheating, we would see the same difference in cheating rates with bankers as well as with students: it would not matter that the banking identity or stereotype are not self-relevant enough for the students and therefore not primed in the same way as for bankers (Shih et al., 2002). However, this experiment was done with a much lower incentive ($5 vs. $20 for each successful guess), which is known to increase overall cheating (Mazar et al., 2008). In addition, students could perceive bankers as an out-group, which may have led them to attempt to distance themselves from it and its expected dishonest behavior and thus lower the cheating (Gino et al., 2009). And in fact, students in the control group really cheated more than did bankers in the control condition (57.9 vs. 51.6% of flips reported as successful), but they also cheated (although not significantly) more than students in the experimental group (57.9 vs. 56.4%), which in any case limits the comparability of the results of these two experiments. Moreover, the underlying assumptions that questions associated with work in a bank activate the concept of money in the same way for bankers as for students is itself questionable and unsupported.

The correct way of ruling out the alternative explanation would be to prime bankers with money (e.g., with images of bills) without making their identity or work-associated social norms salient (which can be tested with the same manipulation check already used by the authors).

There is also a possibility that questions about work invoked feeling of entitlement to financial reward. It is known that more productive workers tend to cheat more (Gill et al., 2013) and bankers, after answering questions about their work, could feel they are good in a very stressful job and thus deserve a greater reward. Other professionals may compare themselves with their peers preferably on other dimensions, but for bankers, we would expect the financial comparison is readily available. However, this will only strengthen the tendency to cheat, as people act more dishonestly when they are comparing themselves to those who are better off (John et al., 2014). Nobody is able to assess whether bankers really did feel financially worse off than their colleagues, because while bankers were asked “How high is your salary in comparison to that of other employees in the same firm?” (CFM, Supplementary Information, p. 29), non-banking professionals got the question with “the national average salary” as a reference for a comparison.

In order to rule out this alternative explanation, the sense of entitlement should be controlled, as it is possible that bankers feel greater entitlement to financial reward than other professionals.

One surprising finding in the CFM paper is that the estimate of cheating behavior did not differ between the experimental and control group. From previous research it is known that unethically behaving people expect others to behave the same (Abbink and Herrmann, 2011), hence we would expect the estimate of cheating to be higher in a group that cheats more, regardless of what caused the increase in cheating. However, these estimates were not obtained from the actual participants in the main study, but from a separate sample of different bankers. They were divided to the experimental or control condition and read the same materials as the participants in the original study. But instead of actually performing the coin flipping task, they were only asked to estimate, with a financial incentive for a correct guess, the number of wins the participants reported on average. Interestingly, participants quite over-estimated the proportion of winning flips in the control condition (57.1 vs. 51.6% in the main study) (CFM, Supplementary Information, p. 13), which suggests that the originally observed result could be anomalous and the main experiment should be replicated with a new sample of bankers.

## Conclusions

With all the above mentioned issues taken together, it becomes clear that there are several plausible alternative explanations for the effect observed in CFM which were not ruled out. Therefore, we believe that the strong conclusions of the CFM study are not fully justified. Especially the claim that social norms in the banking industry encourage dishonesty lacks empirical support, as all results are perfectly in line with the possibility that not norms, but societal expectations are causing the difference in cheating. Moreover, the observed difference could have been caused by priming the control group with free time and/or by priming some other concepts together with the professional identity in the experimental group. All of these alternative explanations may be less sensational than the one chosen by the authors; however, one should always resist the temptation to over-confidently interpret the empirical data.

In any case, the main finding should be replicated with a different sample of bankers, in order to establish dependability of the effect (Brandt et al., 2013)—regardless of its cause(s).

### Conflict of interest statement

The authors declare that the research was conducted in the absence of any commercial or financial relationships that could be construed as a potential conflict of interest.
